# Creation of an Analytical Model of Spinal Cord Cooling by Epidural Catheter for Preventing Paraplegia

**DOI:** 10.7759/cureus.20430

**Published:** 2021-12-15

**Authors:** Syunsuke Masuda, Atsuo Mori, Satoshi Mizonishi, Ryoichi Tashiro

**Affiliations:** 1 New Business Development, Cybernet Systems Co. Ltd., Tokyo, JPN; 2 Cardiovascular Surgery, Kawasaki Municipal Hospital, Kawasaki, JPN; 3 Department of Bioengineering, Saitama Cardiovascular and Respiratory Center, Saitama, JPN

**Keywords:** computer simulation, paraplegia, epidural catheter, cooling, spinal cord ischemia

## Abstract

Introduction

Paraplegia is a serious complication after thoracic and thoracoabdominal aortic aneurysm surgery. The aortic cross-clamp blocks blood flow to the intercostal artery as a feeding blood vessel, so the spinal cord is at risk of being exposed to ischemia. Hypothermia with systemic cooling is a useful means of avoiding spinal ischemia caused by aortic blockade but has various side effects. Theoretically, local cooling by epidural cooling catheter is an effective method to reduce the side effects. However, the use of needle sensors to measure the temperature of the human spinal cord is not ethically applicable in the real clinical field. The purpose of the study is to build computer modeling of human-sized spinal cords and a basic platform for simulating spinal cord cooling. This is being done to prove that local cooling can cool the human spinal cord in the same way, even in human spinal cords larger than laboratory animals.

Methods

We tried to model a horizontal cross-section of tissue near the spinal cord at a size equivalent to that of an adult human. The tissue around the spinal cord was decomposed into many small matrices for analysis using the finite element method. Next, the analysis was performed using a high-speed computer on the assumption that the matrix exchanges heat with the adjacent matrix over time according to Pennes' bio-heat equation. Repeated calculations were performed on a high-speed computer to calculate temperature changes in the central part of the spinal cord.

Result

By setting the temperature of the cooling catheter to 20°C, temperatures at the center of the spinal cord after 5, 10, 15, 20 and 25 minutes were 34.08°C, 33.64°C, 33.48°C, 33.40°C, and 33.36°C, respectively. After stopping the cooling, the temperature at the center of the spinal cord recovered to baseline temperature within 10 minutes.

Conclusion

Results were similar to those of previous animal studies using our local cooling system, suggesting that evaluation of cooling catheter's performance by computational simulation (CS) is effective.

## Introduction

Paraplegia is a serious complication that affects 3-17% of patients after prosthetic graft replacement surgery for thoracic and thoracoabdominal aortic aneurysm [1-4]. The spinal cord receives blood flow mainly from the anterior radicular artery, which is anatomically connected to intercostal arteries or lumbar arteries. The identification of the blood vessel to be reconstructed during surgery is difficult. Aortic cross-clamping associated with prosthetic graft replacement interrupts blood flow in all branches from the clamped segment of the aorta, leading to spinal cord ischemia injury. 

Systemic hypothermia is considered to protect the spinal cord from ischemic stress during aortic cross-clamping by suppressing the metabolic rate and suppressing the oxygen consumption of neurons.

The lower the body temperature, the better the spinal cord can be protected. However, whole-body hypothermia also causes abnormal coagulation of blood, bleeding tendency due to platelet consumption, and bradycardia. As a result, to overcome such disadvantages while retaining the advantages of hypothermia, a strategy involving local cooling of the spinal cord rather than the whole body may represent an ideal solution.

We have proposed a new method for cooling the spinal cord with a catheter to prevent spinal cord ischemia, which we term as the continuous cord cooling (CCC) system. We have developed this method to cool the spinal cord by circulating physiological saline through a dedicated percutaneous catheter and continuously transferring heat from the spinal cord to the catheter [5,6].

Our laboratory is investigating the effects of local cooling using the CCC system in experimental animals. In an animal survival study using pigs, we demonstrated that the CCC system is effective for protecting the spinal cord from ischemic injury. It also allows the animal to withstand the spinal cord ischemia resulting from thoracic descending aortic cross-clamping for up to 30 minutes [5-7]. Furthermore, by applying the CCC system to a rabbit model of spinal cord ischemia, we were able to confirm synergistic protective effects of the CCC system and whole-body cooling with blankets [8].

However, one possible limitation of these experiments is that the animal experiments using pigs and rabbits are insufficient to prove that the CCC system can cool a human-sized spinal cord as it is markedly larger than that of laboratory animals. Since the use of a needle sensor insertion for measuring temperature in the human spinal cord could lead to traumatic injury, including paraplegia/paraparesis, it may not be applicable in clinical practice. To address this obstacle, we have attempted to build a basic platform for computer modeling and simulation of a human-sized spinal cord.

## Materials and methods

Epidural cooling catheter and continuous cord cooling system

**Figure 1:**
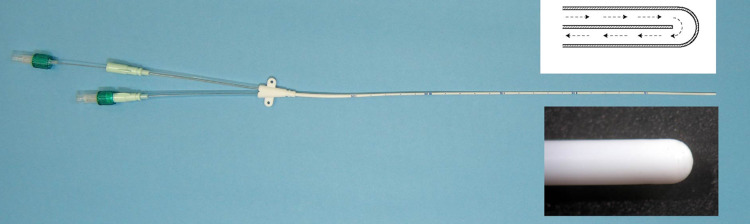
Epidural cooling catheter.

**Figure 2:**
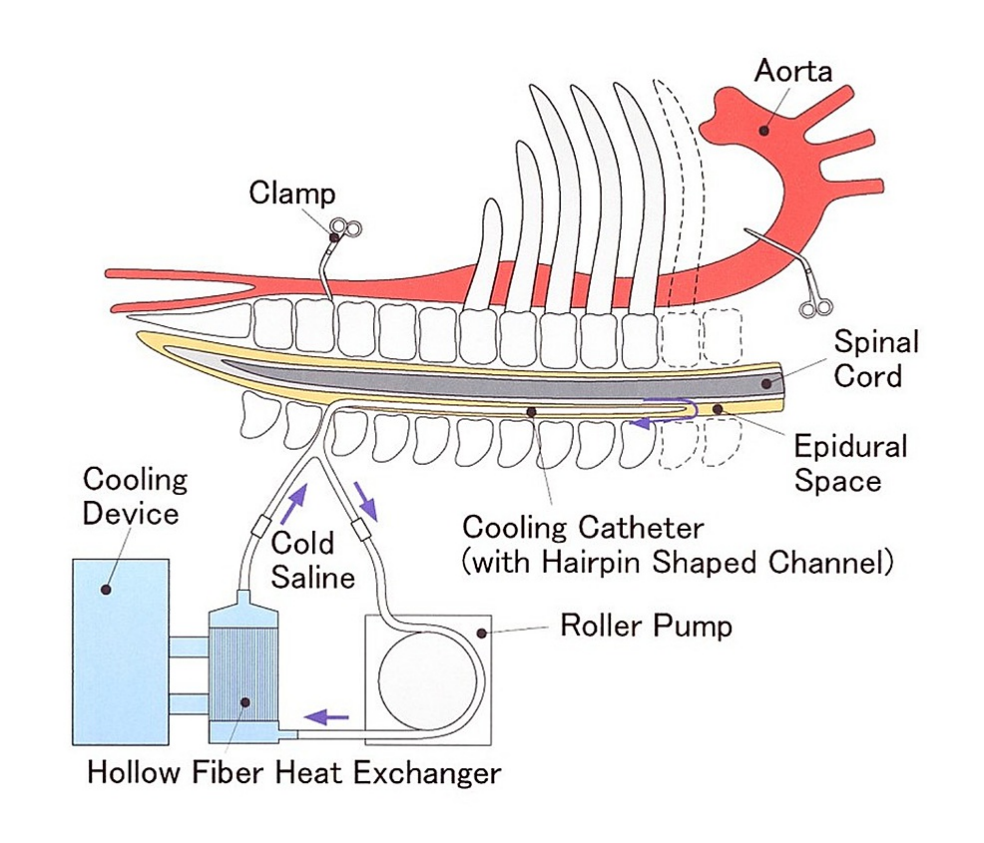
Continuous cord cooling system.

The basic concept of our cooling system has been reported previously [5,6]. We developed a double lumen catheter with a unique U-shaped tip that circulates cooled saline within a closed countercurrent lumen (Figure 1). The outside driving unit comprises a circulating rotator pump as a heat exchanger (Figure 2). Cooled physiologic saline circulates through the circuit, removing heat from the spinal cord continuously.

Calculation model

The computational model involves a two-dimensional model representing the axial cross-section of a spinal cord and tissues around it as a simplified model based on clinical data.

**Figure 3:**
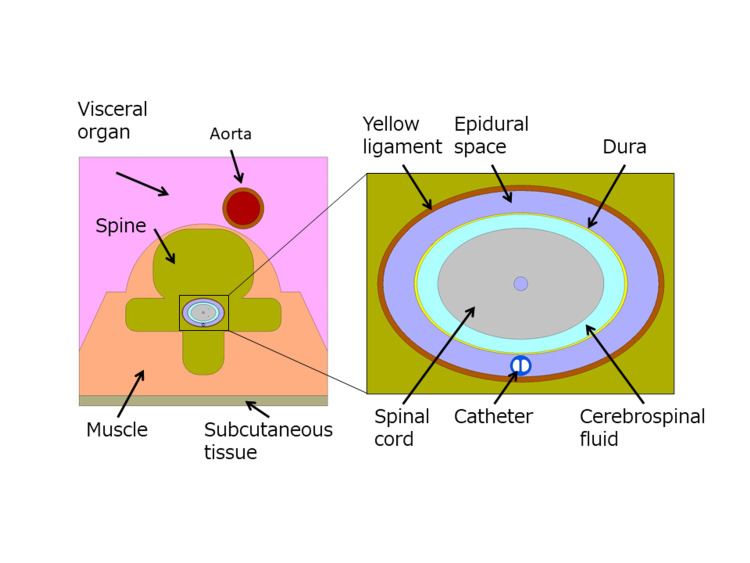
A basic model of tissues near the spinal cord.

Based on CT image data from an adult male, the shape of the region for analysis was created by combining basic geometric figures such as ellipses and rectangles, recreating the structure of the spinal canal, including the vertebral body centered on the spinal cord and surrounding organs. The catheter that cools the spinal cord is placed in the center of the dorsal side of the epidural space as a dual-cavity structure (Figure 3).

A calculation lattice was created for this region shape. The analysis conditions shown in Table 1 were set, then thermal fluid analysis was performed in consideration of cerebrospinal fluid flow and conjugate heat conduction of each tissue. The viscosity of cerebrospinal fluid was set to 0.001 Pa s.

**Table 1:** Material properties.

Tissue	Density (kg/m^3^)	Thermal conductivity (W/[m K])	Specific heat (J/[kg K])
Cerebrospinal fluid	1,100	0.57093	1,000
Spinal cord	1,075	0.51	3,630
Dura	1,174	0.44	3,364
Epidural space	911	0.21	2,348
Yellow ligament	1,142	0.47	3,432
Spine	1,178	2.2093	1,313
Muscle	900	0.49	3,421
Splanchnic tissues	737	0.465	3,749.5
Subcutaneous tissue	1,009.5	0.29	2,869.5
Catheter	900	0.3349	550

In our calculations, the thermal equilibrium state without cooling using the catheter was prepared by stationary analysis. Material properties, boundary conditions (heat transfer condition, flow condition), simulation time, and time step are summarized in Tables 2-4.

The ANSYS 18.2 general-purpose multiphysics analysis tool (ANSYS Inc., Canonsburg, PA) was used for defining the region shape, grid for calculation, and numerical calculations.

**Table 2:** Boundary condition of heat transfer.

Category of condition	Cooling phase	After stoppage of cooling
Catheter temperature (℃)	20, 16, 12, 8	Adiabatic
Heat convection (W/[m^2^ K])	Natural convection (temperature dependence)	Natural convection (temperature dependence)
Ambient temperature (℃)	30	30

**Table 3:** Simulation time.

Category of parameter	Cooling phase	After stoppage of cooling
Analysis time (min)	30	30
Time step (s)	0.02	0.02

**Table 4:** Heat rate condition.

Tissue	Heat rate (W/m^3^)	Circulatory blood flow (kg/s/m^3^)
Spinal cord	2,666	3.01
Dura	6,914.86	7.80710
Epidural space	464.61	0.52610
Yellow ligament	513.9	0.57957
Spine	176.7	0.20615
Muscle	864	0.61425
Visceral organ	14,668.51	10.48333
Subcutaneous tissue	1,146.98	1.29136

Penne's bio-heatequation

Spontaneous fever in each organism and warming by blood flow are represented by the second term (q_p_: perfusion heat by blood) on the right-hand side of the Penne's bio-heat equation shown below, while the third term (q_m_: generated metabolic heat) was considered a heat-generating term in all tissues except the catheter [9].

*ρ* c =∇(k∇T) + q_p_ + q_m_

where *ρ *is specific gravity, c is specific heat, T is temperature, k is thermal conductivity, q_p_ is perfusion heat by blood, and q_m_ is generated metabolic heat.

The net inflow can be written as: q_p_= -ω_b_ρ_b_ c_b_*ρ*(T -Ta) where ω_b_ is the blood perfusion (volume blood per unit mass of tissue per unit time, 1/s), ρ_b_ and c_b_ are the density and specific heat of the blood, and Ta is the temperature of the arterial blood.

## Results

**Figure 4:**
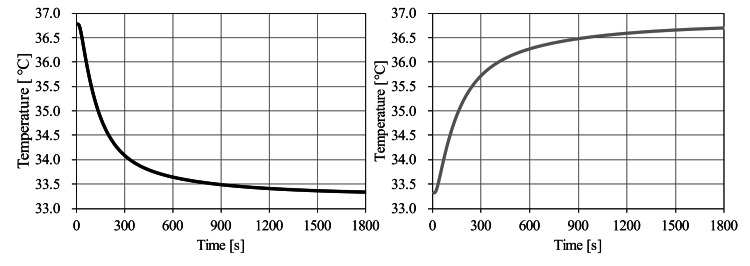
The time course of temperature at the center of the spinal cord.

The temperature of tissues, other than the cooling catheter, at 0 minutes was in thermal equilibrium without cooling by the catheter. The dynamic characteristics of temperature in the vicinity of the spinal cord with the temperature of the cooling catheter set at 20°C are shown in Figure 4. Temperatures at the center of the spinal cord after 2, 5, 10, 15, and 20 minutes were 35.14°C, 34.08°C, 33.64°C, 33.48°C, and 33.40°C, respectively. By stopping the cooling, temperatures of the central portion of the spinal cord after 2, 4, 6, 8, and 10 minutes increased to 34.65°C, 35.49°C, 35.90°C, 36.13°C, and 36.27°C, respectively.

**Figure 5:**
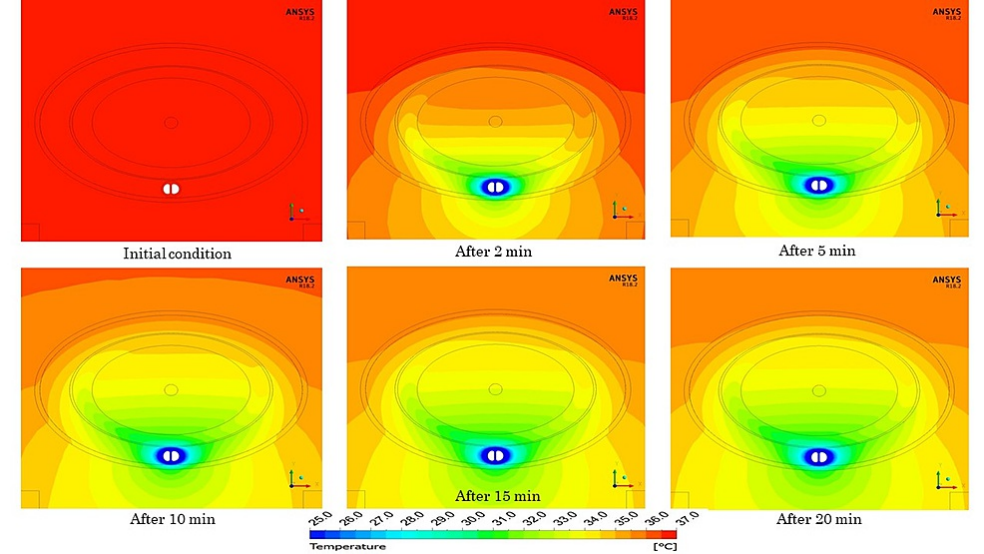
Temperature distribution of cooling phase.

**Figure 6:**
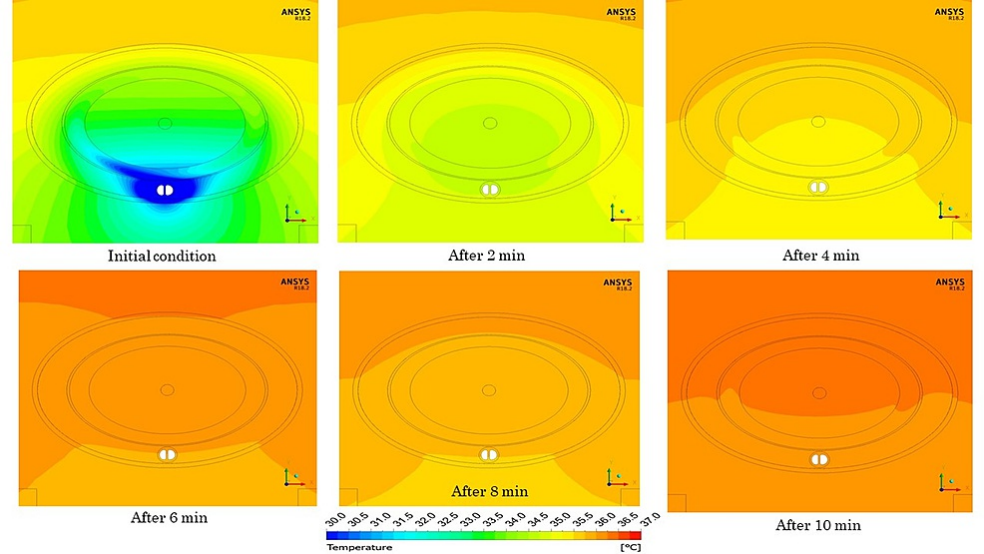
Temperature distribution after the stoppage of cooling.

As for the temperature distribution near the spinal cord, a temperature gradient was formed from the dorsal side to the ventral side, where the cooling catheter was placed (Figure 5). With the passage of time, the cooling effect of the cooling catheter on the surrounding tissues was increased (Figure 6).

## Discussion

The results of measuring the cooling effect of the spinal cord by the CCC system in our computer simulation (including the temperature attained) matched well with results obtained from measuring the spinal cooling performance of the CCC in laboratory animals (pigs and rabbits) using sensors on the spinal cord [5-8]. The pattern of elevation of spinal temperature during the suspension of CCC use was also similar to the results of animal experiments [5-7].

The return of spinal cord temperature to baseline, approximately 10 minutes after the stoppage of the cooling by CCC, suggests that continued circulation of the cooling vehicle is necessary to keep the spinal cord hypothermic.

We have been conducting research using pigs and rabbits in our laboratory for a long time, but these methods encounter problems in terms of the physical size differences between laboratory animals and humans. Our results provide the first evidence that temperature in the spinal cord can be reduced using a cooling catheter in a human-sized environment, just as in smaller experimental animals.

Animal experiments encounter limitations such as differences in weight and height between humans and animals, but computational simulation (CS) allows settings to be changed freely. Changing various parameters related to the conditions around a spinal cord, evaluation of the cooling performance could also be possible in a human-sized spinal cord. 

One key limitation of this research was that the model is not three-dimensional spatial but two-dimensional. The spinal cord is not actually a simple cylindrical structure. A key danger is that simplification associated with such modeling can represent a source of bias. Therefore, without placing too much confidence in the results of CS, analyses to confirm conformation to the results of animal experiments and clinical data are also needed.

However, we clarified that comprehensive analyses performed in silico with due care could provide valuable results such as detailed local information and elucidation of new mechanisms; these could not be clearly elucidated in vivo or in vitro.

## Conclusions

We have built a basic platform for two-dimensional CS of spinal cord cooling. CS has confirmed that spinal cord cooling with an epidural cooling catheter with a closed-circuit can cool even a human-sized spinal cord. This suggests that this cooling method would be useful in avoiding ischemic spinal cord injury associated with aortic surgery. CS is expected to be a promising research tool as a means of thermo-fluid analysis in tissue cooling in the future.
